# Deep-Sea Octopus (*Graneledone boreopacifica*) Conducts the Longest-Known Egg-Brooding Period of Any Animal

**DOI:** 10.1371/journal.pone.0103437

**Published:** 2014-07-30

**Authors:** Bruce Robison, Brad Seibel, Jeffrey Drazen

**Affiliations:** 1 Research Division, Monterey Bay Aquarium Research Institute, Moss Landing, California, United States of America; 2 Department of Biological Sciences, University of Rhode Island, Kingston, Rhode Island, United States of America; 3 Department of Oceanography, University of Hawaii, Honolulu, Hawaii, United States of America; The Evergreen State College, United States of America

## Abstract

Octopuses typically have a single reproductive period and then they die (semelparity). Once a clutch of fertilized eggs has been produced, the female protects and tends them until they hatch. In most shallow-water species this period of parental care can last from 1 to 3 months, but very little is known about the brooding of deep-living species. In the cold, dark waters of the deep ocean, metabolic processes are often slower than their counterparts at shallower depths. Extrapolations from data on shallow-water octopus species suggest that lower temperatures would prolong embryonic development periods. Likewise, laboratory studies have linked lower temperatures to longer brooding periods in cephalopods, but direct evidence has not been available. We found an opportunity to directly measure the brooding period of the deep-sea octopus *Graneledone boreopacifica*, in its natural habitat. At 53 months, it is by far the longest egg-brooding period ever reported for any animal species. These surprising results emphasize the selective value of prolonged embryonic development in order to produce competitive hatchlings. They also extend the known boundaries of physiological adaptations for life in the deep sea.

## Introduction

In April of 2007 we used a remotely operated vehicle (ROV) to inspect an isolated rocky outcrop, 1397 m deep, at the sediment-covered base of a sloping wall in the Monterey Submarine Canyon, off central California. We had visited the site several times before and knew it to be a location where females of *Graneledone boreopacifica* Nesis 1982, attach and brood their eggs. On this occasion we found no brooding individuals on the rock. However, on the sediment nearby we observed a solitary octopus moving slowly toward the exposed hard substrate.

**Figure 1:**
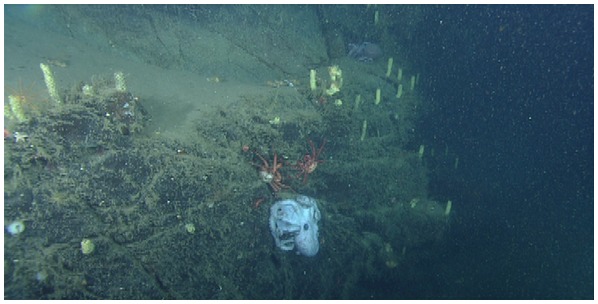
*Graneledone boreopacifica.* The subject female brooding her eggs on a nearly vertical rock face at a depth of 1397*Neptunea amianta*. Near the octopus are two Lithodid crabs and a non-brooding *Graneledone* can be seen above and to the right of the brooder. The mantle length of the specimen, when first encountered, was 21.2 cm.

**Figure 2:**
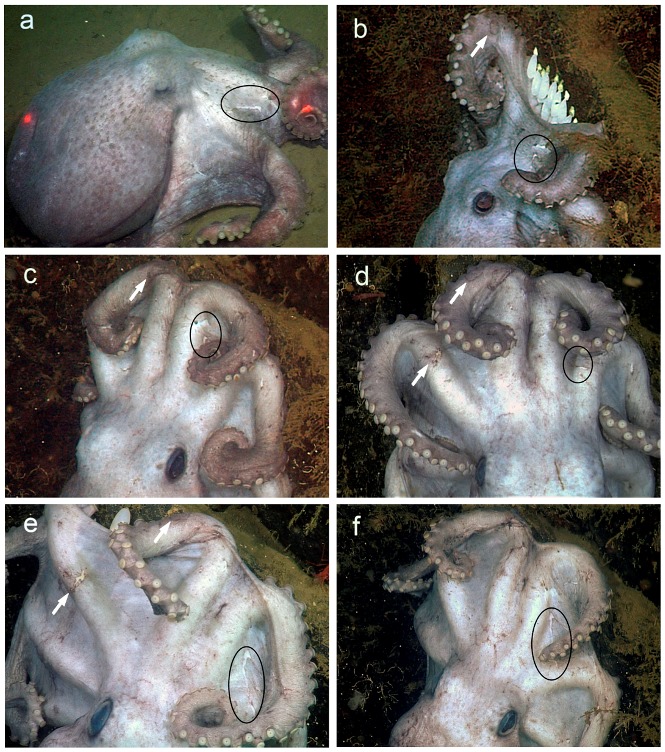
*Graneledone boreopacifica*, identification marks. Images of a brooding female over the course of 53 months, each showing the identifying scar on the web between arms R1 & R2. In each frame the characteristic scar is outlined by an oval. **a,** April, 2007, crawling across the sediment toward the brooding site. **b,** May, 2007, on the rock face, covering the recently deposited clutch of eggs. The arrow points to a circular scar on arm L1, which provides additional confirmation. **c,** May, 2009. **d,** October, 2009. The second arrow points to a scar on arm L2. **e,** December, 2010. **f,** September, 2011.

**Figure 3:**
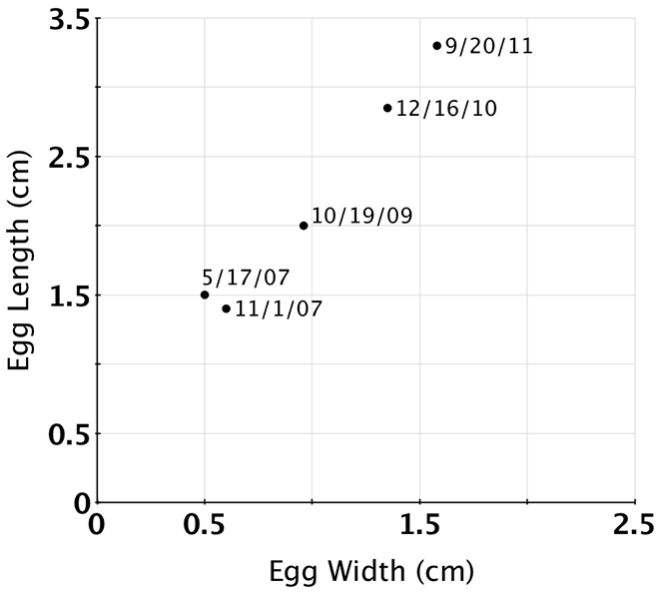
Plot of egg growth over time. The continued growth of the eggs throughout the measurement period indicates that we were observing a single clutch. Because we did not wish to disturb the brooding female, only eggs at the periphery of the clutch were measured.

When we returned to the site 38 days later, in May, 2007, we found the same individual octopus, easily identified by characteristic scars, up on the rock and guarding a clutch of attached eggs (Figure 1). Given this singular opportunity to measure the length of a brooding period from its inception, we returned to the site 18 times over the ensuing four-and-a-half years. Each time we returned we found the same octopus (Figure 2) clinging to the vertical rock face, arms curled, covering her eggs. Continuous growth of the eggs provided evidence that it was the same clutch throughout (Figure 3).

In general, octopods are believed to cease or to greatly reduce feeding when they brood [1]–[4]. For *Graneledone*, there is no evidence that females feed while brooding [5]. Potential prey in the form of Lithodid crabs and Pandalid shrimps were frequently in close attendance around the brooding female that we monitored. However, the only interactions we observed were when she pushed these crustaceans away as they stepped within her watch circle. In laboratory brooding studies, the deep-living octopus *Bathypolypus arcticus* occasionally accepted food that was offered by hand [2]. But in situ, the brooding *Graneledone* ignored pieces of crab when we offered them to her with the manipulator of the ROV.

During the periods when we had the female under observation, she never left the eggs unattended; but our presence included only a small fraction of the protracted brooding period. While the proximity of our ROV was surely conspicuous because of its lights and vibrations, we never disturbed the brooder mechanically. On two occasions we used the reverse water flow of our suction sampler to gently lift the web between her arms so that we could view some of the eggs for laser-referenced size measurements. While she occasionally shifted her position slightly, or uncurled and lifted one or two arms, the female always remained centered over the clutch of eggs.

## Results

**Figure 4:**
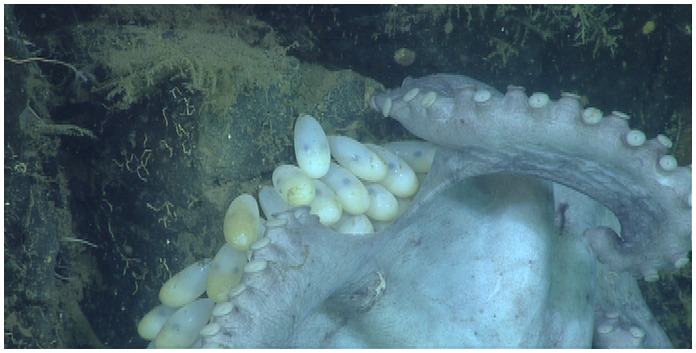
Close-up of the egg capsules in December, 2010. In month 43, the mantles of the embryos can be seen in the apex of each capsule and their dark eyes are apparent.

The eggs of *G. boreopacifica* are elongate vesicles which taper to short stalks that the mother cements to hard substrate [3]. The chorion tissue comprising the vesicle allows the exchange of oxygen and thus maternal care is necessary to prevent fouling and suffocation [6]. Expansion of the chorion during development permits growth [7] but its pliability renders the capsule vulnerable to predation unless the eggs are protected. We measured the size of exposed eggs several times over the course of the brooding period (Figure 3). Egg capsule sizes started at 1.5 cm (length)×0.5 cm (diameter) and reached 3.3 cm×1.6 cm a month before hatching. By the 40th month the shapes of the young octopuses inside, were clearly apparent (Figure 4). Hatchlings of *G. boreopacifica* are the largest and most developmentally advanced coleoid cephalopod hatchlings known to date [7]. They emerge as virtual miniature adults, without a pelagic juvenile phase [7].

When the brooding female was first observed on the seafloor adjacent to the rock outcrop, the skin of her mantle was highly textured and pallid purple in color; when she began brooding it became pale, almost white. Indeed, on several occasions as the ROV approached the rock, the first thing visible was the pale body of the brooder, standing out against the dark background. Signs of advancing senescence [3], [5] included diminishing size and tumescence of the mantle, loss of skin texture, cloudy eyes, slack skin, and a loss of pigmentation on the oral surface of the arms.

**Figure 5:**
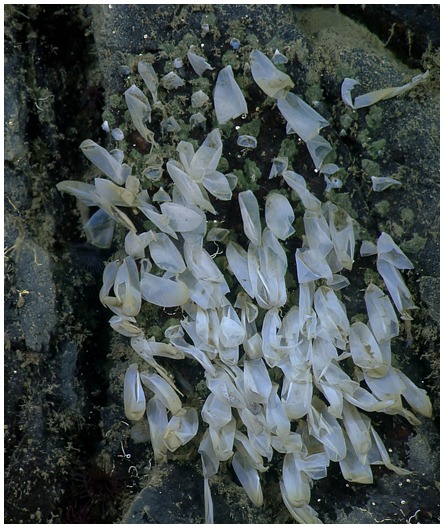
Empty egg cases, October 2011. This is a composite figure, showing empty egg cases, and attachment sites (indicated by green cement residue), used to enumerate the number of egg cases in the clutch, after hatching.

In September of 2011 the brooding female and her eggs were still in place. When we returned in October of 2011 she was gone and the rock face she had occupied held the tattered remnants of empty egg capsules (Figure 5). Thus the total length of the brooding period we measured was from May, 2007 to September, 2011, or 53 months. From HD video frame grabs of the empty capsules and the characteristic green cement of their anchor points [3] we estimate the clutch size to have been between 155 and 165 eggs.

The rock shelf that holds the brooding site runs generally east to west, parallel to the axis of this part of the canyon. The rock face where the octopus placed her eggs is in the lee of sediment flow down the slope of the canyon wall, and parallel to but protected from down-canyon currents. The brooding spot was about one meter above the seafloor sediment and about three meters below the crown of the rocky shelf. We never observed sediment build-up within the small, protected alcove although the canyon is subject to considerable sediment flow [8]. Exposures of hard substrate are rare in this part of the canyon and this rock was occupied by a number of other invertebrate species, both attached and mobile. During the course of our observations the temperature ranged from 2.8°C to 3.4°C.

## Discussion

Aggregations of egg-brooding *G. boreopacifica* have been found elsewhere in the eastern North Pacific, clinging to the vertical faces of rock outcrops where elevated currents reduce sedimentation and promote ventilation of the eggs [3], [9]. In years prior to the present series of observations, we encountered two specimens of *G. boreopacifica* brooding eggs at different locations on the same outcrop in Monterey Canyon. These females were observed in place for 8 months and 20 months but we have no measure of the beginning or end of their full brooding periods. While brooding females are seen routinely at depths between 1200 and 2000 m, we have never observed unattended clutches of *G. boreopacifica* eggs.

Most, if not all incirrate octopods are believed to brood their eggs [7], [10]–[12]. This is part of a holobenthic reproductive strategy that includes direct development without a larval stage. There is considerable variability in reproductive behavior among octopod species from different habitats; but protection of eggs during development is paramount and universal so far as is known. In shallow-water species the eggs are often clustered within a den, where they are guarded and ventilated by the female. In pelagic species, brooding is accomplished with the female holding the developing eggs, usually within her arms. Brooding deep-sea squids also hold their eggs with their arms [13], [14], apparently without feeding. Compared with shallow-water species, the eggs of *G. boreopacifica* are relatively large, while the clutch size is relatively small; both traits are characteristic of a strategy that maximizes the production of developmentally advanced hatchlings [7].

The 53-month brooding period that we measured considerably exceeds all such records in the literature (Table S1 in Information S1). Previously, the longest octopus brooding known was 14 months, attributed to *Bathypolypus arcticus* on the basis of specimens kept in the laboratory [2] at 7°C. The longest guarded incubation known for fish eggs is 4–5 months, by the Magellan Plunder Fish *Harpagifer bispinis* in Antarctic waters [15]. For birds, the longest uninterrupted egg brooding is 2 months, by the Emperor Penguin [16]. Among live-bearing species, elephants gestate for 20 to 21 months [17], frilled sharks carry their embryos internally for about 42 months [18], and the internal gestation period of alpine salamanders can reach 48 months [19] before birth.

The prolonged brooding period that we measured is a consequence of two interacting factors, low temperature, and the selective advantage of producing highly-developed hatchlings. Marine ectotherms typically exhibit an inverse relationship between environmental temperature and the duration of embryonic development [6]. Such a pattern is evident in the semelparous, bathypelagic mysid crustacean *Gnathophausia ingens*, which also has a protracted brooding period of 20 months, hatches miniature adults, and does not feed during brooding [20]. In octopods, development time equates to brooding period and this brings a third factor into play, the brooding parent. In the deep ocean, low temperatures are typical, and thus the trade-off within the reproductive strategy of deep-living octopods is between the mother’s ability to endure a long brooding period, and the competitiveness of her hatchlings. *Graneledone boreopacifica* produces hatchlings that are very highly developed, which gives them the advantage of a high potential for survival [7]. The ultimate fate of a brooding female octopus is invariably death, but in this first example from the deep-sea, brooding also confers an extension of adult life that greatly exceeds most projections of cephalopod longevity.

**Figure 6:**
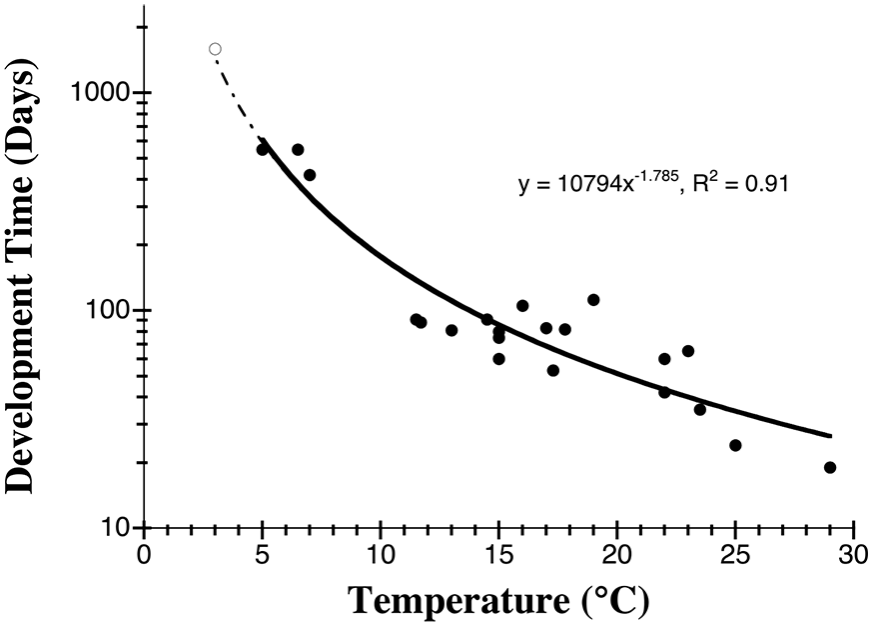
The duration of embryonic development in octopodid cephalopods is related to temperature. Each point represents a different species, measured at the coldest temperature for which data are available (Table S2 in Information S1). The solid line is the best-fit power function for the data from the literature (black symbols). The dashed line extends the model to 3°C, but falls 307 days short of our measured development time for *Graneledone boreopacifica* (open symbol). See also Table S2 in Information S1.

**Figure 7:**
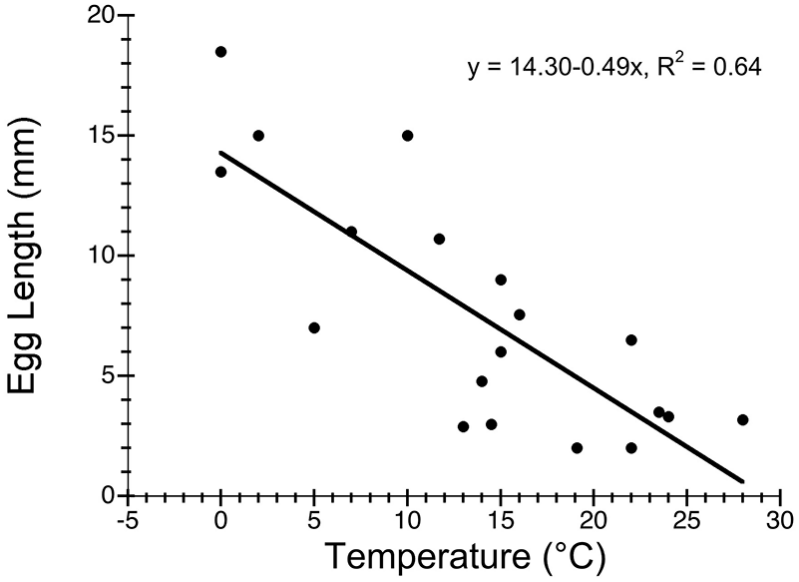
Species living in cold environments have larger eggs. This relationship is, in part, responsible for the longer development times at cold temperatures. It suggests that cold water environments select for better-developed hatchlings and that one mechanism by which octopods achieve this is by increasing egg size. Data from Table S2 in Information S1.

Most previous attempts to model the duration of embryonic development of cephalopods at colder temperatures failed to predict the extended brooding period demonstrated here (Table S1 in Information S1). Interspecific models accounting for variation in egg size and temperature predict anywhere from 2.8 to 75 months for development when applied to *G. boreopacifica*. We show here that much of the interspecific variation in development time can be attributed to temperature (Figure 6). Egg size is also linked to temperature, with longer development yielding larger eggs and more completely developed hatchlings (Figure 7). However, with one exception (Table S1 in Information S1), extrapolation of existing models to 3°C still underestimates measured development in *G. boreopacifica* by nearly a year.

The principal question now remaining is how does the mother survive for so long? Low temperatures and inactivity help by keeping metabolic demand low. We saw no evidence of feeding during brooding. The only examination of a brooding female in the literature showed an empty gut [5]. It is certainly possible that the mother feeds on the surrounding fauna or that in the course of protecting her eggs she feeds on would-be egg predators like Lithodid crabs. Resorption of unlaid eggs is known in other species, as is feeding on unfertilized or diseased eggs [2], [3], [21], [22]. Among deep-sea squids, species that brood their eggs rely on digestive gland lipid stores to meet their extended nutritional needs [13], [14], but octopods are not known to store lipid as extensively. Regardless of how their nutritional needs are met, female *G. boreopacifica* spend a long time brooding. The entire lifespan of most shallow-water cephalopod species is less than 2 years [1], [11]. If *G. boreopacifica* conforms to the general rule among octopods, that brooding comprises about a quarter of the lifespan [10], [23], then it, and possibly other deep-sea species, may be among the longest-lived of all cephalopods [24].

While the discovery of a 53-month brooding period is remarkable, this reproductive strategy is not unusual, it is common and is clearly successful because *G. boreopacifica* is one of the most abundant deep-living octopods in the eastern North Pacific [9], [25], [26]. These results only seem extraordinary when compared with well-studied shallow-water species; which indicates how little we really know about the deep sea.

This study did not involve endangered or protected species. The location of the study site was 36° 42′ N, 122° 03′ W, at a depth of 1397 meters. Field work was conducted under NOAA Office of Marine Sanctuaries permit MBNMS 2005-010-A3, and California Department of Fish and Wildlife scientific collecting permit D-0004069542-0; no specimens were collected.

## Supporting Information

Information S1 **Table S1**, Measured and predicted development time for *Graneledone boreopacifica* using published models. Hatching mass used in some models comes from Voight and Drazen, 2004. Egg length (15 mm) and temperature (3°C) were measured in situ. **Table S2**, Egg development and morphometric data for species of the family Octopodidae.
(DOCX)
