# Supplementary material for: Deep-Sea Octopus (Graneledone boreopacifica) Conducts the Longest-Known Egg-Brooding Period of Any Animal
Source: PLoS One. 2014 Jul 30;9(7):e103437. doi: 10.1371/journal.pone.0103437 (PMC4116195; doi:10.1371/journal.pone.0103437)
Supplement: Information S1 — Table S1, Measured and predicted development time for Graneledone boreopacifica using published models. Hatching mass used in some models comes from Voight and Drazen, 2004. Egg length (15 mm) and temperature (3°C) were measured in situ. Table S2, Egg development and morphometric data for species of the family Octopodidae. (DOCX) [file pone.0103437.s001.docx]

**Table S1.** **Measured (blue shading) and predicted development time (days) for *Graneledone boreopacifica* using published models.** Hatching mass used in some models comes from Voight and Drazen, 2004. Egg length (15 mm) and temperature (3°C) were measured in situ.

| **Reference** | **Model**  **Duration (D, days), Temperature (T,°C)** | **Duration (in days, at 3°C)** | **Notes** |
| --- | --- | --- | --- |
| **This manuscript** | **In situ measurement** | **1590** | **Based on in situ observations with MBARI remotely operated vehicles.** |
| This manuscript | D=10794T^-1.79^ | 1510 | Octopodidae only, each species represented by one point. Where more than one measurement was available, the measurement at the lowest temperature was used. Temperature range = 5-29°C.  **Data in Table S2**. |
| …including *Graneledone boreopacifica* | D=10087T^-1.76^ | 1458 | As above, but including the present data for *G. boreopacifica* at 3°C. |
| Temperature coefficient, Q_10_ = 2 - 3 | R_2_ = R_1_*Q_10_^(T^_2_^-T^_1_^/10)^  Rate (R) = 1/D | 552, 650 | Calculated using a starting development time of 419 days (R_1_ = ^1/^_419_) at 7°C from *Bathypolypus arcticus* (Wood, 1998). |
| Laptikhovsky, 1991 | D=277.6**d*^0.0291T+0.263^e^-0.22T^  d = (L + W/2) x 10 = 100  L = 15, W = 5 | 743 | For Octopodidae. Nesis (1999) used this formula to estimate *G. boreopacifica* embryonic development at 826 days (2.2°C, L = 16, W = 7). An error in Nesis’ formula resulted in 798 days. The formula presented here is correct. |
| Laptikhovsky, 1999 | D=(3163.1T^-1.62^)L^0.499^ | 2057 | For Octopodidae. The author uses “ripe egg length”, which is 35 mm in *G. boreopacifica*. This results in over-estimated embryonic development duration. |
| Seibel et al., 2000 | D=70.67L^-0.86^ | 725 (5-7°C) | Data from varied squids and octopods measured between 5-7°C (n=7). |
| Katsanevakis and Verriopoulos, 2006 | D=532.2/(T-8.76), re-plotted with power function to extrapolate <9°C, D=22391T^-2.023^ | 2425 | Effect of temperature on *Octopus vulgaris,* range 11-31°C |
| Hamasaki and Morioka, 2002 | D=62798T^-2.477^ | 4131 | Effect of temperature on *Octopus vulgaris*, range 16.5-25.5°C- |
| Kubodera, 1991 | 2300 - 2700 deg-days | 760 - 900 | Effect of temperature on *Octopus dofleini,* range 7-16°C |
| Gillooly et al., 2002 | Log_e_ D/m^0.25^=-0.12{T/(1+T/273)}+6.06 | 362 | Based on Metabolic Theory of Ecology, using data for “aquatic ectotherms”. Prediction using 2.5 g, hatchling mass. |
| Hirst and Lopez-Urrutia, 2006 | Log_e_ D/m^0.25^=-0.11{T/(1+T/273)}+6.49 | 572 | Based on Metabolic Theory of Ecology as above, using data for Teuthoidea (squids), 2.5 g hatchling mass. |

**Table S2.** **Egg development and morphometric data for species of the family Octopodidae.**

| **Species** | **Egg length (mm)** | **T (°C)** | **Development Duration (days)** | **Reference** |
| --- | --- | --- | --- | --- |
| *Graneledone boreopacifica* | 15 | 3 | 1590 | This study |
| *Octopus dofleini* | 7 | 5 | 547 | Kubodera, 1991 |
| *Octopus bimaculoides* | 2 | 18 | 82 | Forsythe and Hanlon, 1988 |
| *Octopus joubini* | 3.3 | 22 | 42 | Forsythe and Toll, 1991 |
| *Octopus tetricus* | 2 | 17.3 | 53 | Joll, 1978 |
| *Octopus aegina* | 3.18 | 28 | 19 | Ignatius and Srinivasan, 2005 |
| *Octopus laqueus* | 2.6 | 24 | 25 | Kaneko et al., 2006 |
| *Octopus digueti* | 6 | 15 | 60 | Hochberg (in Mangold et al., 1971) |
| *Octopus rubescens* | 3.0 | 14.5 | 91 | Osborn, 1995 |
| *Octopus maorum* | 6 | 15 | 80 | Anderson, 1999 |
| *Octopus micropyrsus* | 9 | 15 | 75 | Hochberg (in Mangold et al. 1971) |
| *Octopus briareus* | 12 | 23 | 65 | Hanlon, 1977 |
| *Octopus vulgaris* | 2 | 17 | 83 | Caveriviere et al., 1999 |
| *Octopus tehuelchus* | 11 | 19 | 112 | Iribarne, 2009 |
| *Hapalochlaena lunulata* | 3.5 | 23.5 | 35 | Overath and Boletzky, 1974 |
| *Hapalochlaena maculosa* | 6.5 | 22 | 60 | Overath and Boletzky, 1974 |
| *Robsonella fontanianus* | 4.77 | 14 | 93 | Gonzalez et al., 2008 |
| *Robsonella australis* | 2.9 | 13 | 81 | Brought, 1965 (in Mangold et al., 1971) |
| *Eledone cirrhosa* | 7.55 | 16 | 105 | Mangold et al., 1971 |
| *Bathypolypus arcticus* | 11 | 7 | 419 | Wood, 1998 |
| *Enteroctopus megalocyathus* | 10.05 | 11 | 168 | Uriarte et al., 2014 |
| *Paroctopus conispadiceus* | 15 | 6.5 | 307 | Ito, 1983 |
| *Megaledone setebos* | 18.5 | 0 |  | Collins and Rodhouse, 2006 |
| *Pareledone harissoni* | 13.5 | 0 |  | Collins and Rodhouse, 2006 |

**Supporting Table References**

Anderson TJ (1999) Morphology and biology of *Octopus maorum* Hutton 1880 in Northern New Zealand. Bull Mar Sci 65: 657-676.

Caveriviere A, Domain, F, Diallo A (1999) Observations on the influence of temperature on the length of embryonic development in *Octopus vulgaris* (Senegal). Aquat Living Resources 12: 151-154.

Collins MA, Rodhouse PGK (2006) Southern Ocean Cephalopods. Adv Mar Biol 50: 192-265.

Forsythe JW, Hanlon RT (1988) Effect of temperature on laboratory growth, reproduction and life span of *Octopus bimaculoides*. Mar Biol 98: 369-379.

Forsythe JW, Toll RB (1991) Clarification of the western Atlantic Ocean pygmy octopus complex: the identity and life history of *Octopus joubini* (Cephalopoda: Octopodinae). Bull Mar Sci 49: 88-97.

Gillooly JF, Charnov EL, West GB, Savage VM, Brown JH (2002) Effects of size and temperature on developmental time. Nature 417 :70-73.

Gonzalez ML, Arriagada SE, Lopez DA, Perez MC (2008) Reproductive aspects, eggs and paralarvae of *Robsonella fontanianus* (d’Orbigny, 1834). Aquacult Res 39: 1569-1573.

Hamasaki K, Morioka T (2002) Effects of temperature on egg incubation period, and paralarval survival and growth of common Octopus, *Octopus vulgaris* reared in the laboratory. Suisanzoshoku 50: 407-413.

Hanlon RT (1977) Laboratory rearing of the Atlantic reef octopus, *Octopus briareus* Robson, and its potential for mariculture. Proc World Mariculture Soc 8: 471-482.

Hirst A, Lopez-Urrutia A (2006) Effects of evolution on egg development time. Mar Ecol Prog Ser 326: 29-35.

Ignatius B, Srinivasan M (2005) Embryonic development in *Octopus aegina* Gray, 1849. Current Sci 91: 1089-1092.

Iribarne OO (2009) Life history and distribution of the small south-western Atlantic octopus, *Octopus tehuelchus*. J Zool 223: 549-565.

Ito H (1983) Some observations on the embryonic development of *Paroctopus conispadiceus* (Mollusca: Cephalopoda). Bull Hokkaido Reg Fish Res Lab 48: 93-105.

Joll LM (1978) Observations on the embryonic development of *Octopus tetricus* (Mollusca: Cephalopoda). Aust J Mar Freshwater Res 29: 19-30.

Kaneko N, Oshima O, Ikeda Y (2006) Egg brooding behavior and embryonic development of *Octopus laqueus* (Cephalopoda: Octopodidae). Molluscan Res 26: 113-117.

Katsanevakis S, Verriopoulos G (2006) Modelling the effect of temperature on hatching and settlement patterns of meroplanktonic organisms: the case of the octopus. Scientia Marina 70: 699-708.

Kubodera T (1991) Distribution and abundance of the early life stages of octopus, *Octopus dofleini* Wülker, 1910 in the North Pacific. Bull Mar Sci 49: 235-243.

Laptikhovsky VV (1991) A mathematical model for the study of the duration of embryogenesis in cephalopods. Biol Nauki 3: 37-48.

Laptikhovsky VV (1999) Improved mathematical model to study the duration of embryogenesis in cephalopod mollusks. Ruthenica 9: 141-146.

Mangold K, Boletzky Sv (1973) New data on reproductive biology and growth of *Octopus vulgaris.* Mar Biol 19: 7-12.

Mangold K, Boletzky Sv, Frösch D (1971) Reproductive biology and embryonic development of *Eledone cirrosa* (Cephalopoda: Octopoda). Mar Biol 8: 109-117.

Nesis KN (1999) The duration of egg incubation in high-latitude and deep-sea cephalopods. Biolog Morya Vladivostok 25: 499-506.

Osborn SA (1995) Fecundity and embryonic development of *Octopus rubescens* Berry from Monterey Bay, California. MS Thesis. San Jose State University.

Overath H, Boletzky Sv (1974) Laboratory observations on spawning and embryonic development of a blue-ringed Octopus. Mar Biol 27: 333-337.

Seibel BA, Hochberg FG, Carlini DB (2000) Life history of *Gonatus onyx* (Cephalopoda: Teuthoidea): deep-sea spawning and post-spawning egg care. Mar Biol 137: 519-526.

Uriarte I, Espinoza V, Gutierrez R, Zuniga O, Olivares A, Rosas C, Pino S, Farias A (2014) Key aspects of egg incubation in Patagonian red octopus (*Enteroctopus megalocyathus*) for cultivation purposes. Aquaculture 424-425: 158-166.

Voight JR, Drazen JC (2004) Hatchlings of the deep-sea octopus *Graneledone boreopacifica* are the largest and most advanced known. J Moll Stud 70: 406-408.

Wood JB (1998) Reproduction and embryonic development time of *Bathypolypus arcticus*, a deep-sea octopod. Malacologia 39: 11-20.
